# Gastric myoelectric activity during cisplatin-induced acute and delayed emesis reveals a temporal impairment of slow waves in ferrets: effects not reversed by the GLP-1 receptor antagonist, exendin (9-39)

**DOI:** 10.18632/oncotarget.21859

**Published:** 2017-10-16

**Authors:** Zengbing Lu, Man P. Ngan, Ge Lin, David T.W. Yew, Xiaodan Fan, Paul L.R. Andrews, John A. Rudd

**Affiliations:** ^1^ School of Biomedical Sciences, The Chinese University of Hong Kong, Hong Kong SAR, China; ^2^ Brain and Mind Institute, Faculty of Medicine, The Chinese University of Hong Kong, Hong Kong SAR, China; ^3^ Department of Statistics, The Chinese University of Hong Kong, Hong Kong SAR, China; ^4^ Division of Biomedical Sciences, St George's University of London, London, UK

**Keywords:** cisplatin, GLP-1 receptors, gastric myoelectric activity, ferret, emesis

## Abstract

Preclinical studies show that the glucagon-like peptide-1 (GLP-1) receptor antagonist, exendin (9-39), can reduce acute emesis induced by cisplatin. In the present study, we investigate the effect of exendin (9-39) (100 nmol/24 h, i.c.v), on cisplatin (5 mg/kg, i.p.)-induced acute and delayed emesis and changes indicative of ‘nausea’ in ferrets. Cisplatin induced 37.2 ± 2.3 and 59.0 ± 7.7 retches + vomits during the 0-24 (acute) and 24-72 h (delayed) periods, respectively. Cisplatin also increased (*P*<0.05) the dominant frequency of gastric myoelectric activity from 9.4 ± 0.1 to 10.4 ± 0.41 cpm and decreased the dominant power (DP) during acute emesis; there was a reduction in the % power of normogastria and an increase in the % power of tachygastria; food and water intake was reduced. DP decreased further during delayed emesis, where normogastria predominated. Advanced multifractal detrended fluctuation analysis revealed that the slow wave signal shape became more simplistic during delayed emesis. Cisplatin did not affect blood pressure (BP), but transiently increased heart rate, and decreased heart rate variability (HRV) during acute emesis; HRV spectral analysis indicated a shift to ‘sympathetic dominance’. A hyperthermic response was seen during acute emesis, but hypothermia occurred during delayed emesis and there was also a decrease in HR. Exendin (9-39) did not improve feeding and drinking but reduced cisplatin-induced acute emesis by ~59 % (*P*<0.05) and antagonised the hypothermic response (*P*<0.05); systolic, diastolic and mean arterial BP increased during the delayed phase. In conclusion, blocking GLP-1 receptors in the brain reduces cisplatin-induced acute but not delayed emesis. Restoring power and structure to slow waves may represent a novel approach to treat the side effects of chemotherapy.

## INTRODUCTION

The treatment of cancer with cisplatin–based therapies is documented to be associated with the side effects of nausea and emesis (retching and vomiting). Current treatment guidelines include combining 5-hydroxytryptamine_3_ (5-HT_3_) receptor (e.g. ondansetron, palonosetron) and tachykinin neurokinin_1_ (NK_1_) receptor antagonists (e.g. aprepitant, netupitant) with a glucocorticoid, such as dexamethasone [1]. However, not all patients are completely protected [2]. Investigations into the contribution of novel mechanisms to the emetic reflex are required to identify pathways resistant to conventional anti-emetic therapy [3].

5-HT_3_ and tachykinin NK_1_ receptors can be found in brain areas and afferent systems involved in emesis control including the area postrema (AP), nucleus tractus solitarius (NTS) and abdominal vagal neurones, representing logical sites to target drugs to inhibit emesis [3]. Glucagon-like peptide-1 (GLP-1) receptors are also distributed in the same pathways [4, 5], but also in more rostral brain areas (e.g. amygdala, hypothalamus) [4–6], linked to a modulation of biomarkers associated with nausea including plasma vasopressin [7]; gastric myoelectric activity (GMA) [8, 9]; and heart rate variability (HRV) [10]. Not surprisingly, GLP-1 receptor agonists including exenatide (synthetic exendin-4) and liraglutide (used for the treatment of type 2 diabetes) can cause nausea and emesis in man [11].

Rodents are incapable of emesis [12] but have been used to identify sites of action of GLP-1 agonists to induce gastric malaise indicating the involvement of forebrain and hindbrain systems (see below). The effect of the GLP-1 receptor agonist, exendin-4, to cause pica (the consumption of a substance without nutritional value, which is presumed to be an index of emesis) in rats is not prevented by abdominal vagotomy [13], although activation of GLP-1 receptors in the NTS mediate reduction in food intake [14]. Interestingly, the GLP-1 receptor antagonist, des-His^1^-Glu^9^-exendin-4, attenuates LiCl-induced conditioned taste avoidance and anorexia by mechanisms involving the AP, NTS, and lateral parabrachial nucleus [15, 16] demonstrating several sites of action within the brain to modulate malaise and aversive mechanisms.

Studies using *Suncus murinus* (house musk shrew) indicate that intracerebroventricularly (i.c.v.) administered exendin-4 is associated with emesis and an inhibition of food and water intake, reduced locomotor activity, and increases of c-Fos expression in the AP and NTS, hypothalamus and amygdala [17]. Only the emetic effect of exendin-4 and increases of c-Fos in the brainstem and hypothalamus were reversed by blocking GLP-1 receptors with the GLP-1 receptor antagonist, exendin (9-39) [17]. Further studies demonstrated that exendin-4-induced emesis in *Suncus murinus* is not prevented by ondansetron, or the NK_1_ receptor antagonist, CP-99,994, suggesting that GLP-1 receptor pathway(s) involved in emesis and feeding may be resistant to the conventional anti-emetics used in the treatment of chemotherapy-associated side effects [5].

The ferret has been used extensively for research into novel approaches to anti-emesis, particularly in relation to anti-cancer chemotherapy and to identify the emetic liability of novel chemical entities; in both settings the findings have had translational value [18–21]. We have reported previously, using the ferret, that the hypoglycaemic and anorectic effects of exendin-4 administered peripherally (100 nmol/kg, s.c.) were associated with a reduction of HRV and when emesis occurred, it was associated with effects on GMA manifested as bradygastria and reduced power [5], consistent with changes occurring during nausea in man induced by other stimuli [10, 22, 23]. We also showed that exendin-4 administered i.c.v. had similar effects to s.c. exendin-4 on GMA and HRV, but additionally hypertension and tachycardia also occurred [24]. The pattern of c-Fos activation following i.c.v. exendin-4 indicated that emesis is reliant on the brainstem, but ‘behaviours indicative of nausea’ most likely involve more rostral brain nuclei [25].

Exendin (9-39) administered i.c.v. antagonises the early emesis and associated increase in c-Fos in the dorsal vagal complex and the paraventricular hypothalamus induced by cisplatin in *Suncus murinus* [5]. However, no studies using a species capable of emesis have investigated the anti-emetic potential of GLP-1 receptor antagonists against both acute and delayed phases of cisplatin-induced emesis. Therefore, the present study was designed to investigate the anti-emetic potential of exendin (9-39) infused *continuously* using the well-characterised ferret model of acute and delayed emesis induced by cisplatin [26]. In addition to emesis, using radiotelemetry, the effects of cisplatin alone or with exendin (9-39) on GMA, cardiovascular function, body temperature, food and water intake were also recorded to access data that may be relevant to mechanisms underlying nausea [24, 25].

It is clear that GMA recordings have a visually apparent ‘slow-wave’ structure that changes in humans reporting nausea [23] and in animals given emetic stimuli [25, 27, 28]. However, these features may be lost during conventional signal processing techniques (e.g. fast Fourier transformation; FFT) to produce an electrogastrogram. Therefore, we applied multifractal detrended fluctuation analysis (MFDFA) to the telemetric recordings to examine if slow wave shape had been changed following cisplatin [29]. Fractal analyses have been used in other areas of signal processing to differentiate between healthy and pathological conditions (e.g. [30, 31]). Hence, we applied MFDFA to analysis of the GMA to more fully characterise the gastric effects of cisplatin during the acute and delayed phase of emesis and the involvement of GLP-1 receptors.

## RESULTS

### General comments

Recovery of animals from surgery was generally uneventful and they ate and drank normally within the first day following the implantation of transmitters. However, one animal assigned to the Ex-9 + Cisplatin treatment group developed a suspected bladder infection, so it was excluded from the study. An implanted transmitter malfunctioned in an animal that was assigned to the Ex-9 + Cisplatin treatment group and therefore its telemetric data could not be recorded.

### Effect of an i.c.v. infusion of exendin (9-39) (100 nmol/24 h) or saline (10 μl/h) on cisplatin (5 mg/kg, i.p.)-induced acute and delayed emesis

**Figure 1:**
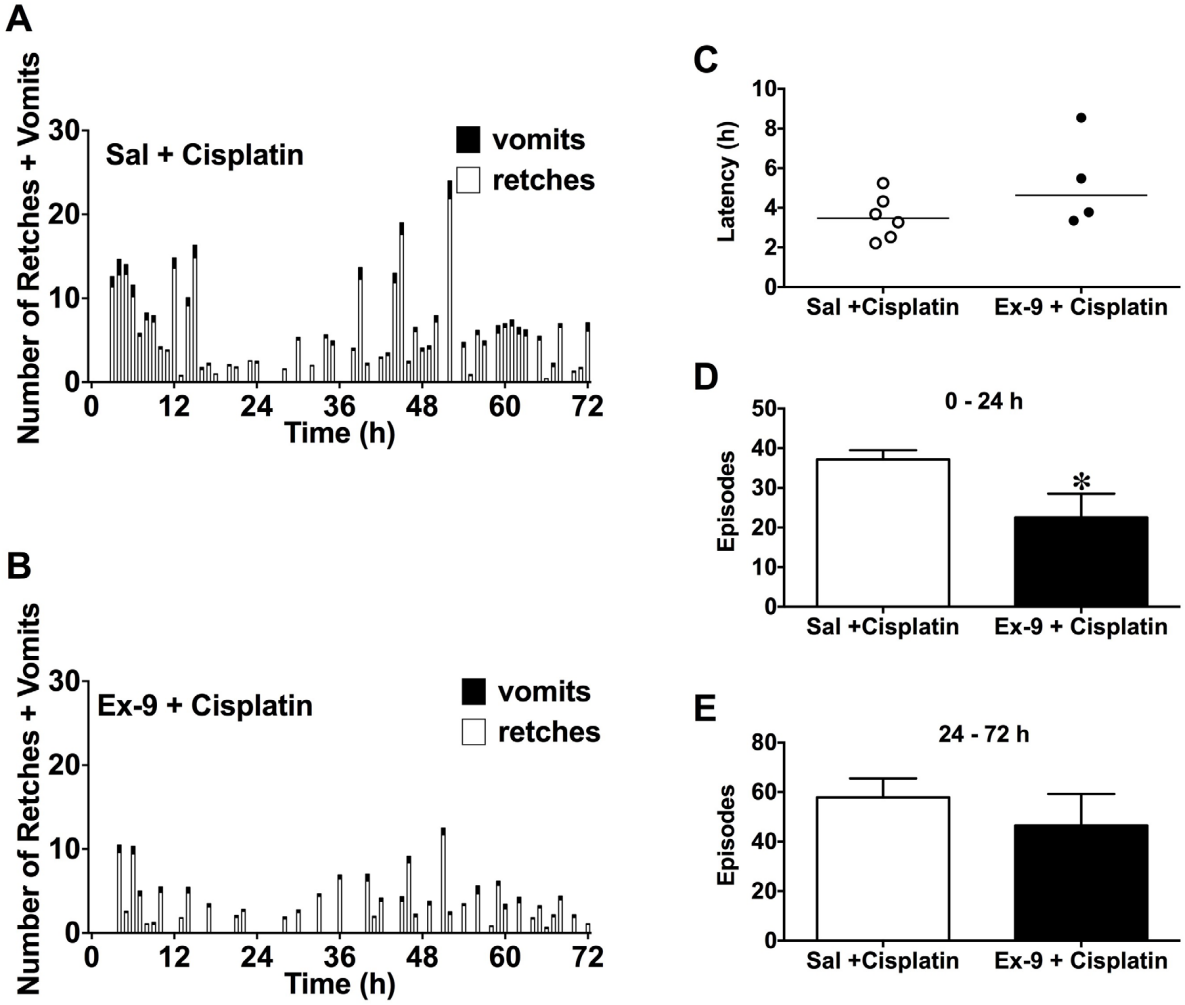
Effect of an i.c.v. infusion of saline (10 μl/h) or exendin (9-39) (100 nmol/24 h) on emesis induced by cisplatin (5 mg/kg, i.p.) Profiles of emesis are shown in **(A)** and **(B)**; latency to the first emetic episode **(C)**; number of episodes of emesis during the 0-24 h (acute phase) **(D)** and 24-72 h (delayed phase) **(E)** are also shown. Data represents the mean ± SEM of 4-6 animals. Sal = saline, Ex-9 = exendin (9-39). Significant differences between Sal and Ex-9 groups are shown as ^*^P<0.05 (unpaired t-test).

In the vehicle-treated animals, cisplatin induced emesis following a median latency of 3.5 h (range 2.2-5.2 h). The response occurring during the acute (0-24 h) phase consisted of 37.2 ± 2.3 episodes and comprised 246.7 ± 29.3 retches and 26.3 ± 0.8 vomits. The response occurring during the delayed (24-72 h) phase consisted of 59.0 ± 7.7 episodes and comprised 372.8 ± 59.4 retches and 38.8 ± 6.2 vomits. The infusion of exendin (9-39) did not affect the latency to the first episode (exendin (9-39)-treated animals, median latency = 4.6 h; range 3.4-8.5 h; *P*>0.05) but did significantly reduce the number of episodes of retching and/or vomiting, and number of retches + vomits during the acute phase by 39.5 and 59.3 %, respectively (*P*<0.05; Figure 1); the number of retches was not affected (a non-significant 43.3 % reduction was recorded; *P*>0.05, Figure 1). Exendin (9-39) did not affect the number of episodes or the number of retches or vomits during the delayed phase (modest 15-26 % non-significant reductions were observed; *P*>0.05, Figure 1).

### Effect of an i.c.v. infusion of exendin (9-39) (100 nmol/24 h) or saline (10 μl/h) on GMA in animals treated with cisplatin (5 mg/kg, i.p.)

**Figure 2:**
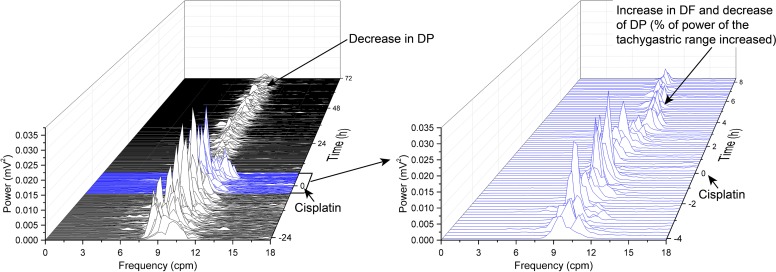
Running spectrum analysis (RSA) of gastric myoelectric activity (GMA) in the vehicle-control ferret that received cisplatin (5 mg/kg, i.p.) **(A)** RSA of GMA from start of baseline recording 24 h prior to cisplatin administration to 72 h after cisplatin administration; **(B)** an expanded view of the RSA from 4 h before cisplatin injection to 8 h post-cisplatin injection. DF = dominant frequency, DP = dominant power. This animal exhibited emesis after a latency of 2.52 h; it had 13 episodes of emesis during the first 8 h period (during the 0-24 h and 24-72 h periods, it had 37 and 26 episodes of emesis, respectively). Note the decrease in DP and increase in DF following cisplatin administration (arrowed boxes).

**Figure 3:**
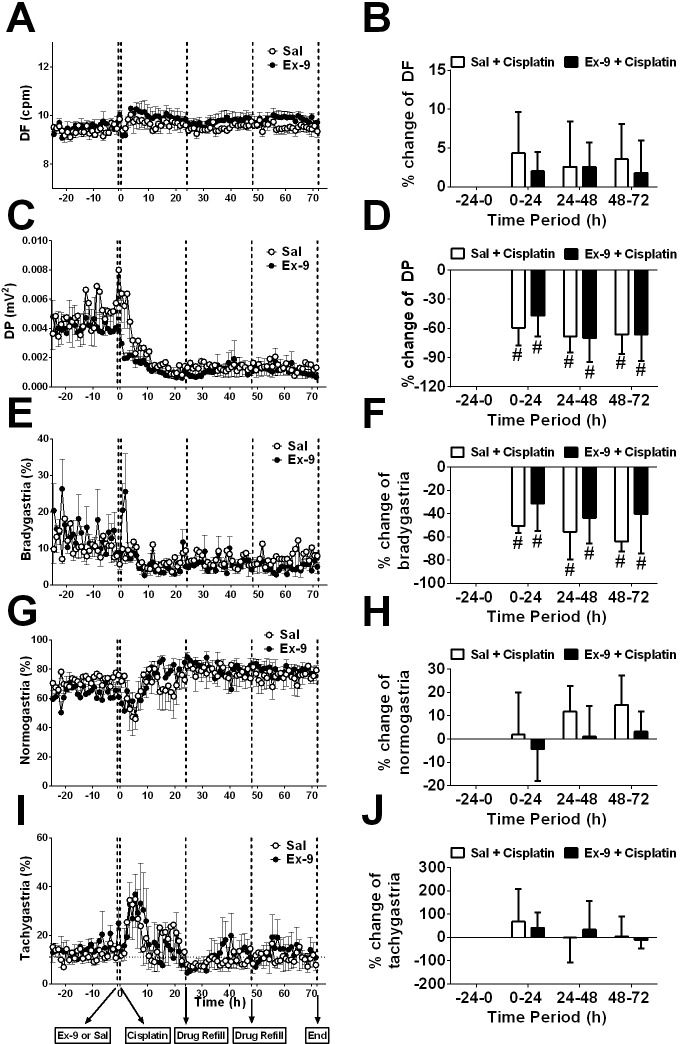
Effect of an i.c.v. infusion of saline (10 μl/h; open circles) or exendin (9-39) (100 nmol/24h; filled circles) on gastric myoelectric activity in animals given cisplatin cisplatin (5 mg/kg, i.p.) **(A)** DF, **(B)** percentage change of DF over 24 h, **(C)** DP, **(D)** percentage change of DP over 24 h, **(E)** bradygastria (%), **(F)** percentage change of bradygastria over 24 h, **(G)** normogastria (%), and **(H)** percentage change of normogastria over 24 h, **(I)** tachygastria (%), and **(J)** percentage change of tachygastria over 24 h are shown. Sal = saline, Ex-9 = exendin (9-39), DF = dominant frequency, DP = dominant power. Data represents the mean ± SEM of 4-6 animals. Significant differences relative to baseline recordings of the respective treatment groups are shown as ^#^*P*<0.05 (one-way ANOVA).

**Figure 4:**
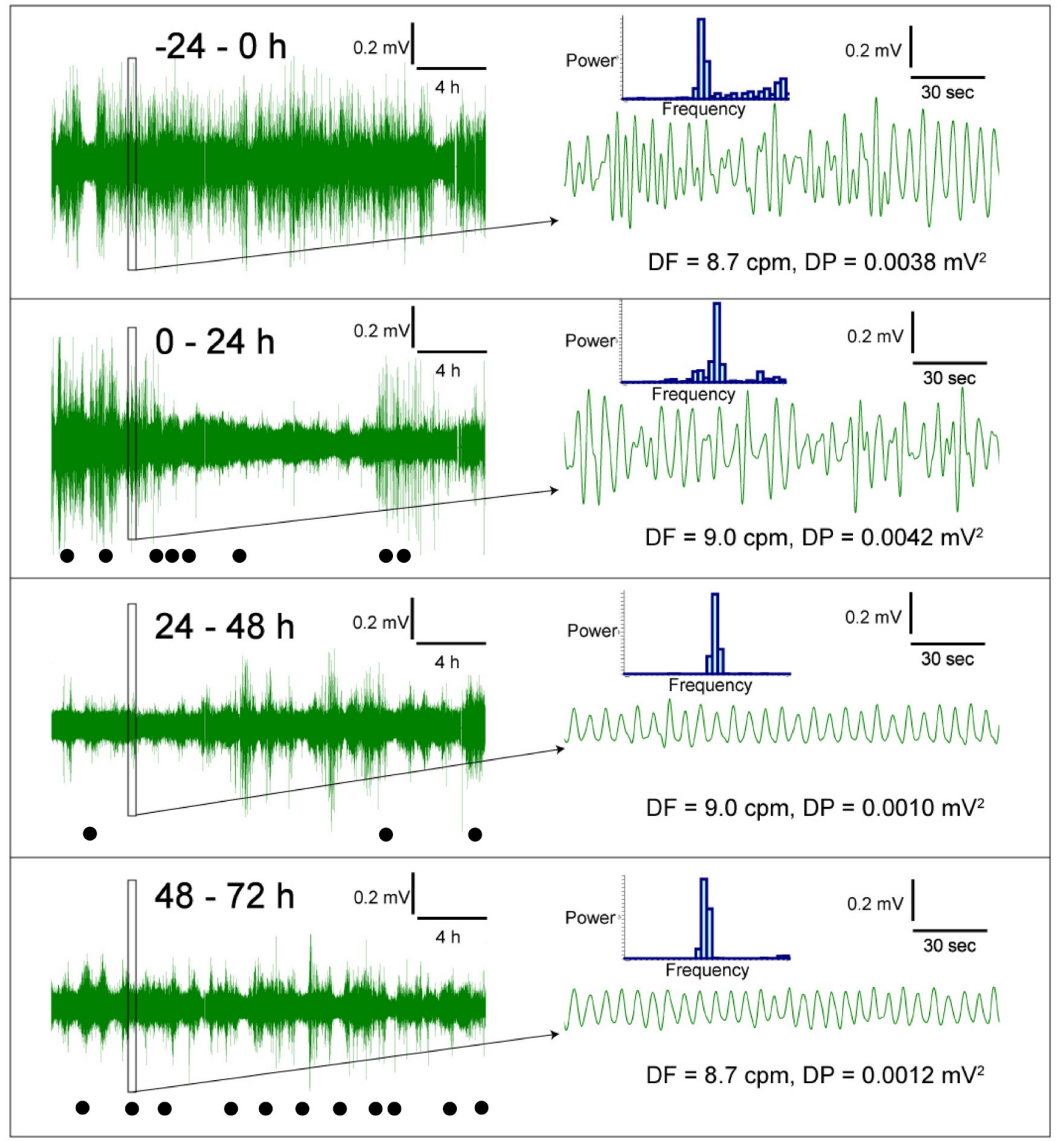
Representative traces of gastric myoelectric activity recordings in a ferret that had received cisplatin (5 mg/kg, i.p.) This animal exhibited emesis after 2.52 h. Filled dots indicate episodes of retching and/or vomiting during 1 h period.

Prior to randomization to the treatment groups, the baseline GMA recordings (-24-0 h) revealed a dominant frequency (DF) of 9.4 ± 0.1 cpm; 12.1 ± 0.5% of power was in the bradygastric range, 68.4 ± 0.5% of power was in the normogastric range, and 13.0 ± 0.4% of power was in the tachygastric range (pooled data, n = 10). In the saline infusion control group, cisplatin caused a transient increase in DF (to ~ 10.8 cpm) and a 50 % decrease in dominant power (DP) during the first 8 h following cisplatin injection; there was with a 35.9 % decrease in the % power of normogastria and a 167.2 % increase in the % power of tachygastria (*P*<0.05; n = 6, Figure 2 and Figure 3). After 12 h, when the initial acute phase of emesis had begun to subside there was a decrease in DP (to ~ 0.003 mV^2^), with a 74.5 % decrease in the % power of bradygastria (*P*<0.05; n = 6, Figure 3 and Figure 4); normogastria (~80 %) then dominated for the remainder of the recording period, and the % power of tachygastria was ~10 %. The i.c.v. infusion of exendin (9-39) failed to affect the changes in GMA caused by cisplatin when analysed using conventional statistical approaches (*P*>0.05; n = 4-6, Figure 3 and Figure 4).

**Figure 5:**
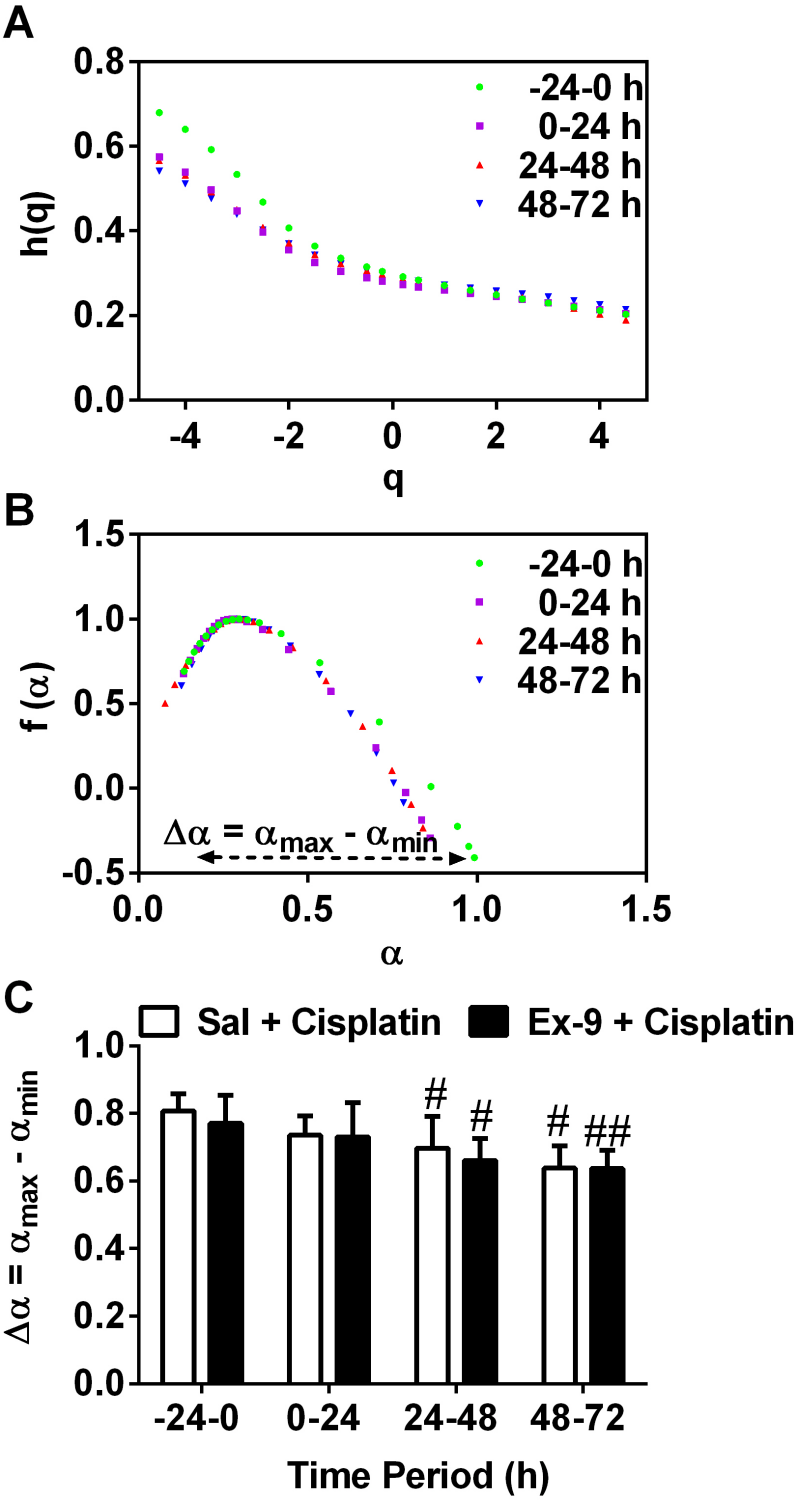
Multifractal detrended fluctuation analysis (MFDFA) **(A)** Generalized Hurst exponent h(q) *vs*. order q, and **(B)** dimension of subset series f(α) *vs*. Singularity strength α for gastric myoelectric activity in one ferret during four different time periods: pre-cisplatin(-24-0 h) and post-cisplatin-Sal (0-24 h, 24-48 h, 48-72 h); **(C)** the width of the multifractal spectrum (Δα) of four different periods (-24-0 h, 0-24 h, 24-48 h, 48-72 h) in Sal and Ex-9 are shown, and data represents the mean ± SEM of 4-6 animals. Sal = saline, Ex-9 = exendin (9-39). Significant differences relative to baseline recordings of the respective treatment groups are shown as ^#^*P*<0.05, ^##^*P*<0.01; no significant differences were found between Sal and Ex-9 groups (one-way ANOVA or two-way ANOVA as appropriate).

Application of MFDFA to the same data sets revealed that the baseline (-24-0 h) data had a multifractality magnitude of 0.791 ± 0.02 (arbitrary units). In the saline infusion group, cisplatin injection had no effect on multifractality during the 0-8, or 0-24 h periods (*P*>0.05). However, cisplatin decreased significantly the magnitude of the multifractality in the 24-48 h and 48-72 h periods, by 13.6 and 21.0 %, respectively (*P*<0.05; n = 4-6, Figure 5). The i.c.v. infusion of exendin (9-39) failed to affect the changes of multifractality magnitude induced by cisplatin (*P*>0.05; n = 4-6, Figure 5).

### Effect of an i.c.v. infusion of exendin (9-39) (100 nmol/24 h) or saline (10 μl/h) on bodyweight, food and water intake, in animals treated with cisplatin (5 mg/kg, i.p.)

Before drug administration, body weight was 1.53 ± 0.07 kg and food and water intake was 33.46 ± 2.78 and 80.21 ± 9.06 g/kg (pooled data, n = 10), respectively. In the saline group, cisplatin reduced body weight (-4.85 ± 0.54 % at t = 24 h, -8.89 ± 0.48 % at t = 48 h and -13.39 ± 0.69 % at t = 72 h), food (-88.15 ± 2.20 % at 0-24 h, -98.6 ± 1.34 % at t = 24-48 h and -100 % at t = 48-72 h) and water intake (-74.11 ± 8.52 % at t = 0-24 h, -88.22 ± 4.76 % at t = 24-48 h and -83.90 ± 9.16 % at t = 48-72 h; *P*<0.05; n = 6, Table 1); these reductions were not prevented by exendin (9-39) (*P*>0.05; n =4-6, Table 1).

**Table 1:** Effect of an i.c.v. infusion of saline or exendin (9-39) (100 nmol/24 h) on body weight, food intake and water intake when animals received cisplatin

	Body weight (% change)		Food intake (% change)		Water intake (% change)	
	Sal + Cisplatin	Ex-9 + Cisplatin	Sal + Cisplatin	Ex-9 + Cisplatin	Sal + Cisplatin	Ex-9 + Cisplatin
Baseline	0 ± 0%	0 ± 0%	0 ± 0%	0 ± 0%	0 ± 0%	0 ± 0%
0-24 h	-4.85 ± 0.54%^###^	-5.12 ± 1.04%^###^	-88.15 ± 2.19%^###^	-83.99 ± 2.98%^###^	-74.11 ± 8.52%^###^	-67.37 ± 4.68%^###^
24-48 h	-8.89 ± 0.48%^###^	-9.57 ± 1.27%^###^	-99.66 ± 1.34%^###^	-98.66 ± 0.98%^###^	-88.22 ± 4.76%^###^	-79.87 ± 8.40%^###^
48-72 h	-13.39 ± 0.70%^###^	-14.75 ± 1.92%^###^	-100 ± 0%^###^	-100 ± 0%^###^	-83.90 ± 5.16%^###^	-85.11 ± 5.53%^###^

Data represents the mean ± SEM of 4-6 animals. Sal = saline, Ex-9 = exendin (9-39). Significant differences relative to baseline of the respective treatment groups are shown as ^###^*P*<0.001 (one-way ANOVA).

### Effect of an i.c.v. infusion of exendin (9-39) (100 nmol/24 h) or saline (10 μl/h) on cardiovascular homeostasis and body temperature in animals treated with cisplatin (5 mg/kg, i.p.)

**Figure 6:**
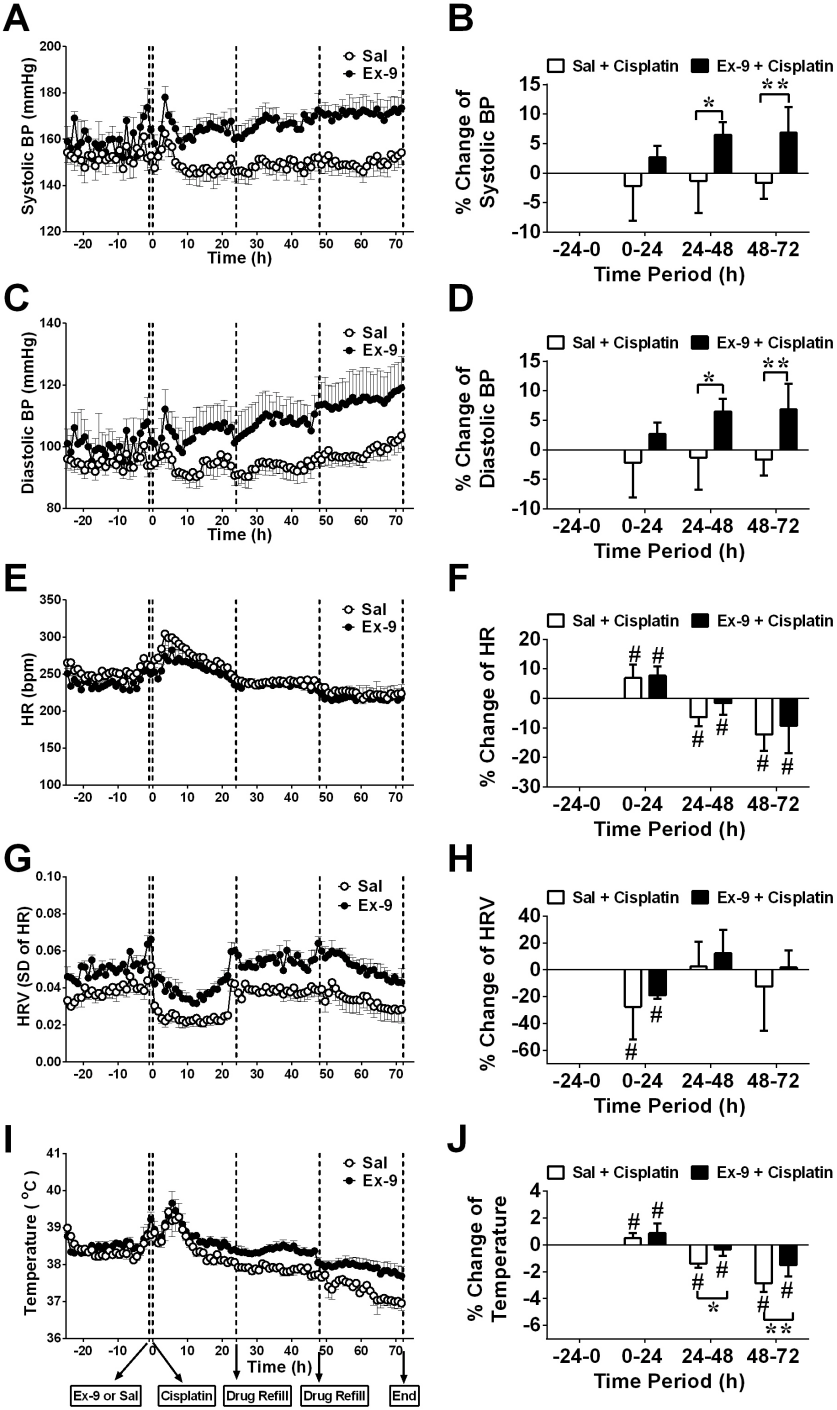
Effect of an i.c.v. infusion of saline (10 μl/h; open circles) or exendin (9-39) (100 nmol/24 h; closed circles) on systolic blood pressure (BP), diastolic BP, heart rate (HR), heart rate variability (HRV) and core body temperature when animals received cisplatin (5 mg/kg, i.p.) **(A)** Systolic BP, **(B)** percentage change of systolic BP over 24 h, **(C)** diastolic BP, **(D)** percentage change of diastolic BP over 24 h, **(E)** HR, **(F)** percentage change of HR over 24 h, **(G)** HRV, **(H)** percentage change of HRV over 24 h, **(I)** core body temperature, **(J)** percentage change of core body temperature over 24 h are shown. Sal = saline, Ex-9 = exendin (9-39). Data represents the mean ± SEM of 4-6 animals. Significant differences relative to baseline recordings of the respective treatment groups are shown as ^#^*P*<0.05; significant differences between Sal and Ex-9 groups are shown as ^*^P<0.05, ^**^P<0.01 (one-way ANOVA or two-way ANOVA as appropriate).

**Figure 7:**
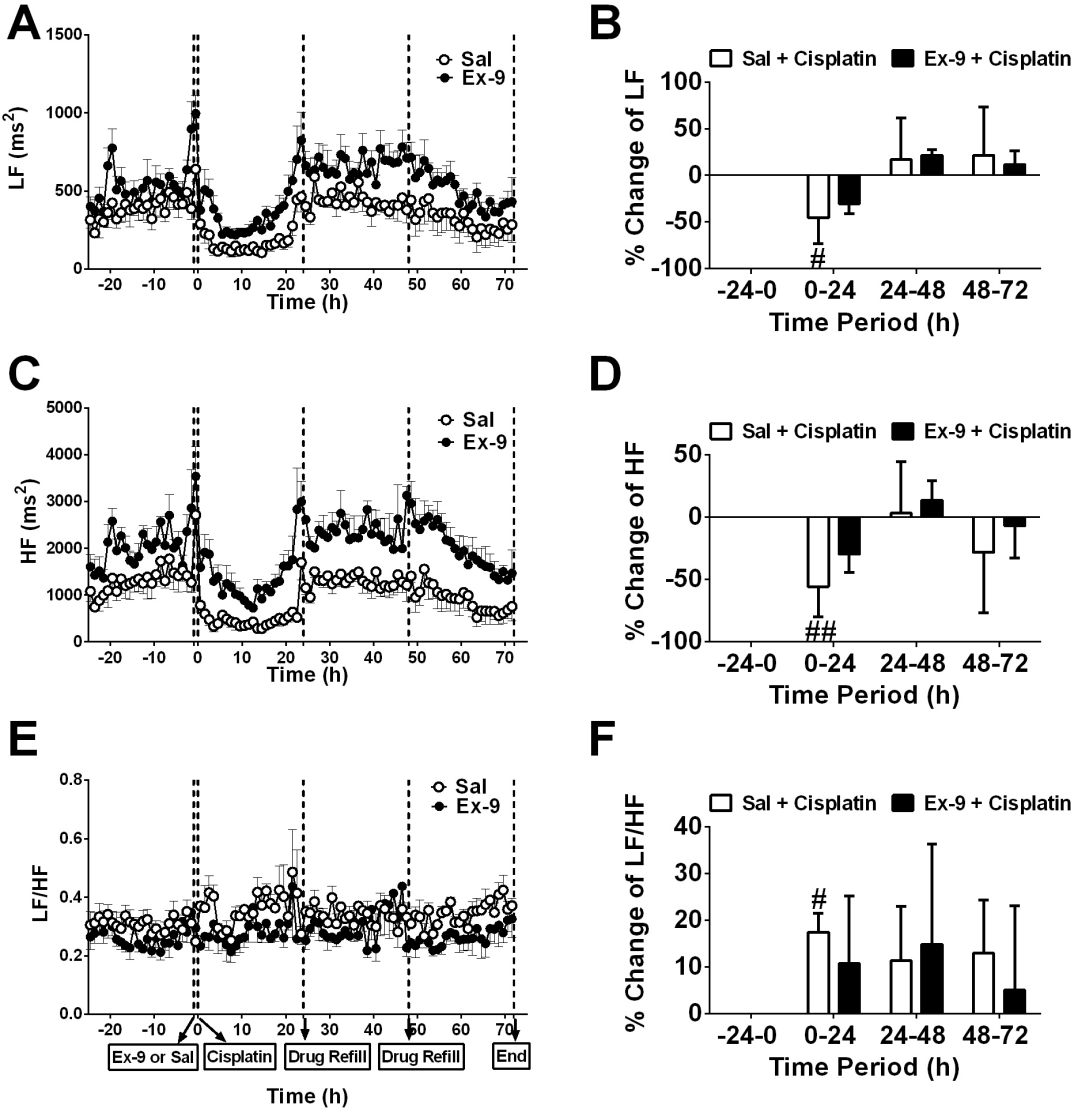
Effect of an i.c.v. infusion of saline (10 μl/h; open circles) or exendin (9-39) (100 nmol/24 h; closed circles) on heart rate variability (HRV) when animals received cisplatin (5 mg/kg, i.p.) **(A)** Low frequency (LF), **(B)** percentage change of LF over 24 h, **(C)** high frequency (HF), **(D)** percentage change of HF over 24 h, **(E)** LF/HF, **(F)** percentage change of LF/HF over 24 h are shown. Sal = saline, Ex-9 = exendin (9-39). Data represents the mean ± SEM of 4-6 animals. Significant differences relative to baseline recordings of the respective treatment groups are shown as ^#^*P*<005 (one-way ANOVA or two-way ANOVA as appropriate).

During baseline recordings (-24-0 h), arterial blood pressure (BP) was 116.8 ± 0.6 mmHg (systolic BP 155.6 ± 0.7 mmHg; diastolic BP 97.3 ± 0.5 mmHg; pulse pressure: 58.3 ± 0.3 mmHg) (pooled data, n = 10); heart rate (HR) and HRV were 249.6 ± 1.3 bpm and 0.0408 ± 0.0008 (arbitrary units), respectively (pooled data, n = 10); core body temperature (CBT) was 38.4 ± 0.1°C (pooled data, n = 10). In the saline group, cisplatin injection caused a transient increase in HR and CBT (an increase of ~1.9°C was observed; *P*<0.05) with a ~30 % decrease in HRV during the first 8 h following cisplatin administration (*P*<0.05; n = 6, Figure 6); there was also an increase in HR (~ up to 50 bpm) and a decrease in HRV (a ~ 25 % fall) during the acute phase of emesis (0-24 h) (*P*<0.05; n = 6, Figure 6). As for HRV, a more detailed analysis showed that compared to baseline (0-24 h prior to cisplatin: low frequency (LF) = 394.9±15.9 ms^2^; high frequency (HF) = 1347.0±73.2 ms^2^, and LF/HF = 0.3079±0.005, arbitrary that cisplatin caused significant falls in LF (~ 40% fall; *P*<0.05) and HF (~ 55% fall; *P*<0.01) translating to an overall 20 % increase in the LF/HF ratio during the acute phase but not delayed phase (Figure 7). During the delayed phase there was a decrease in HR and also a decrease in CBT (~ 1.5°C fall) compared with baseline recordings at t = -24-0 h (*P*<0.05; n = 6, Figure 6). There were no major effects of cisplatin treatment on BP (*P*>0.05; n = 6, Figure 6). The i.c.v. infusion of exendin (9-39) had no effect on any of the parameters recorded during the acute (0-24 h) phase, but caused an increase in systolic BP, diastolic BP and mean arterial BP during the delayed (24-72 h) phase compared with saline group (*P*<0.05, Figure 6). Exendin (9-39) failed to prevent the changes in HR and HRV (including LF, HF and LF/HF) caused by cisplatin (*P*>0.05; n = 4-6; Figure 6 and Figure 7). However, exendin (9-39) partially prevented the hypothermia caused by cisplatin compared with saline group during the delayed phase (*P*<0.05; n = 4-6, Figure 6I and 6J).

## DISCUSSION

This study is the first to characterise GMA, cardiovascular parameters (HRV, BP) and temperature throughout both the acute and delayed phases of cisplatin-induced emesis in an animal model with proven translational value [19]. Cisplatin clearly affected both the power and frequency of gastric slow waves and applying the MFDFA for the first time to analyze the gastric effects of a cancer chemotherapy agent revealed that the magnitude of the multifractality of the structure of the slow waves was reduced in the delayed phase. The present investigations are also the first to reveal the differential action of the GLP-1 receptor antagonist, exendin (9-39), to reduce acute but not delayed emesis induced by cisplatin. The findings are discussed below in detail together with the related effects on core body temperature and the cardiovascular system.

### Cisplatin induced emesis

The acute emetic response to cisplatin in the ferret is primarily by a release of 5-HT from intestinal enterochromaffin cells and activation of 5-HT_3_ receptors on the peripheral terminals of abdominal afferents projecting to the NTS (for reviews including evidence from other species [32]). It is during the initial phase of cisplatin-induced emesis that i.c.v. exendin (9-39) reduced emesis by ~40 % indicating involvement of central GLP-1 receptors in the emetic response to this cancer chemotherapeutic agent. GLP-1 receptors in the brainstem (AP and NTS) are implicated in the emetic response [17, 25]. Previous acute studies in *Suncus murinus* showed that exendin (9-39) antagonised the early emetic response to cisplatin and also the associated increases of c-Fos expression in the NTS, AP, dorsal motor nucleus of the vagus nerve, and paraventricular hypothalamus [17]. Activation of abdominal vagal afferents by cisplatin is the most likely mechanism by which the central GLP-1 pathways are activated in this acute phase. In the ferret, the peripherally acting emetic agents LiCl and cholecystokinin both activated GLP-1-positive neurones in the NTS [33]. In addition, in rats there is evidence that stimulation of 5-HT_3_ receptors located on abdominal vagal afferents drive the release of GLP-1 in approximately 20-30 % of neurons in the NTS following gastric distension [34]. The reduction of the acute emetic response by ~59% with exendin (9-39) implies that either the dose used was not fully efficacious, despite using an infusion, or more likely that other central transmitters such as substance P are also implicated (for review see [35]).

### Cisplatin and gastric myoelectric activity

In conscious ferrets during short duration recording, cisplatin decreased the % power of normogastria (~26 %) and increased the % power of bradygastria, without modifying DF; these changes were most apparent during the first 2-3 h of 4 h total recording time and were associated with periods of emesis [28]. A study in conscious dogs showed that cisplatin (1.2 mg/kg, i.v.) interrupted gastric and intestinal inter-digestive myoelectric activity with the latency for onset of myoelectric changes being ~11 min less than for the onset of emesis [36] and the myoelectric changes lasting for at least 24 h. Another dog study found that cisplatin (1.5 mg/kg, i.v.) decreased by 22 % the time that GMA frequency was in the normal 4-6 per min range (methods to determine DF and associated parameters were not clearly defined) leading the investigators to conclude that dysrhythmia had occurred during their 6 h recording session [27]; however, this conclusion was based on a simplistic method of analysis, only assessing a shift to bradygastria or tachygastria, without assessing the power of the slow waves, or if the slow waves were in fact ‘disordered’. A similar study in dogs showed an increase in gastric dysrhythmia for a period of 6 h following cisplatin [37].

We initially used conventional analytical techniques (FFT, power partitioning) to establish the effects of cisplatin 5 mg/kg, i.p. on GMA. We found that cisplatin induced an increase in DF, with increases in tachygastria during the first 8 h; this was balanced by a corresponding decrease in normogastria. In contrast to the acute phase emesis, the GMA changes were unaffected by central exendin (9-39) indicating that unlike emesis the GMA response to cisplatin does not involve central GLP-1 receptors.

During the delayed emesis phase (24-72 h), conventional analytical techniques did not reveal further changes in the DF of the GMA: the power partition for normogastria predominated, suggesting that cisplatin had no effect on gastric slow wave rhythm during the delayed phase of emesis. However, MFDFA of 24 h epochs of data revealed a reduction in the magnitude of the multifractality indicative of reduced complexity in the form of the slow wave and an increased regularity. We were initially cautious to not over interpret the findings, since this is the first study where MFDFA has been applied to GMA. Yet the differences in slow wave structure were qualitatively visually apparent and did not appear to involve noise.

The antral electrodes are capable of detecting extra-antral signals so digital off-line filtering was applied between 0-0.3 Hz (0-18 cycles per min (cpm)). GMA manifests as slow waves is a summation of voltages coming from interstitial cells of Cajal (ICC; [38, 39]) and smooth muscle [40]. Gastric slow wave can also be modulated by stretch [41], temperature [42], hormones (e.g. vasopressin [43]), and the autonomic system [39] via effects on the ICCs and smooth muscle. Whilst there is no evidence for a direct action of cisplatin on ICCs or smooth muscles, in humans cisplatin can cause cardiac arrhythmia with a direct effect on cardiac sodium channels implicated [44]. In isolated dorsal root ganglion neurones, cisplatin in the clinical concentration range (1-10 μM) decreased input conductance and increased excitability [45]. Evidence was presented for effects of cisplatin on voltage activated K^+^ and Ca^++^ currents and Ca^++^ activated Cl^-^ conductance [46]. Taking these observations together with the data on the diversity of conductance in ICCs [47], we hypothesise that cisplatin has a direct effect on ICCs leading to a simplification of their structure (reflected in the MFDFA). However, as the major effect of cisplatin on GMA revealed by MFDFA occurred in the delayed phase, it is possible that by this time damage is occurring to the enteric nervous system and is responsible for the effects. In rats treated with a single dose of cisplatin, gastric iNOS, L-citrulline and Ca^++^ calmodulin complex were reduced by 3 days [48] and in mice treated with oxaliplatin both morphological and neurotransmitter (increase NOS-IR) changes within 3 days of administration [49].

Clearly, cisplatin can disrupt digestive tract myoelectric activity which is likely to impact contractile activity of the gastrointestinal tract. As the relationship between the GMA signal (frequency or power) and contractile activity of the gastric antrum is not clearly defined [50, 51], we are unable to predict the gastric motility patterns in either the acute or the delayed phases following cisplatin. Recordings of gastric contractile activity following cisplatin are rare; in the dog [52], studies showed disruption of the inter-digestive motor complex (6 h recording) and jejuno-gastric retrograde contractions associated with emesis. Studies of the acute effect of cisplatin on gastric emptying have only been undertaken in rodents where a marked, dose-related, delay is reported consistently [53].

### Cardiovascular effects of cisplatin and GLP receptors

In patients, treatment with cisplatin also causes an elevation of HR and decreases HRV during the first 24 h, suggesting a change in autonomic outflow [54]. A link between HRV and nausea has been demonstrated by several studies [10, 22, 54]. In this study, the decrease in HRV and the shift to “sympathetic dominance” (trend to increase the LF/HF ratio; faster heart rate) occurred mainly in the acute phase of cisplatin-induced emesis. As a general biomarker of ‘nausea’, we would have expected HRV to be clearly affected during both phases, but this was not the case. This difference in the autonomic outflow to the heart between the acute and delayed phase may reflect the shift in the underlying mechanisms driving emesis from an abdominal vagal afferent dominant pathway to the area postrema [28]

Cisplatin-induced vascular toxicities such as endothelial injury, myocardial infarction and a reduced baroreflex sensitivity have been found after acute chemotherapy [55] and this may have posed a problem, or compromised the interpretation of HRV data (above). Nevertheless, BP normally elevates transiently before the onset of retching and/or vomiting, but when data were averaged into one hour time periods, cisplatin had no overall effect on systolic or diastolic BP during acute emesis in the ferret; this is consistent with data from the study of emesis during cisplatin-based chemotherapy schedules in man [54, 56]. Our long-duration recordings extended this knowledge to show that cisplatin also does not change BP during the delayed phase of emesis. However, HR did decrease, indicating that impaired baroreceptor reflex functioning persists into the delayed phase of emesis. However, activation of GLP-1 receptors has been shown to previously increase BP and HR and decrease HRV in ferrets [5] and rats [57, 58]. Further, pre-treatment with exendin (9-39) has been shown to antagonise the effect of GLP-1 on BP and HR in rats [57]. Yet, the present studies found that exendin (9-39) significantly elevated blood pressure in cisplatin-treated animals. Unfortunately, we did not examine the effect of exendin (9-39) administered alone so we do not know if BP was affected by exendin (9-39) alone, or was the result of an interaction between exendin (9-39) and cisplatin. Evidence from other studies showed exendin (9-39) had no effect on BP: an acute i.c.v. injection or i.v. infusion of exendin (9-39) at 2500 ng did not alter arterial BP or HR in rats [57], and i.v. infusion of exendin (9-39) in healthy human subjects had no effect on BP [59]. As exendin (9-39) infusion did not have significant effects on the first 24 h of recordings, it is possible that long-term infusion caused accumulation of exendin (9-39) in the brain, which might induce a supra-physiological effect on the BP. It is possible, therefore, that the change in BP might be due to the combined effect of antagonism of GLP-1 receptors and the potential cisplatin-induced cardiovascular complications (see above).

### Temperature

In the present study, cisplatin also induced hyperthermia over 8 h post cisplatin, and this has not been reported previously, but related studies implicate inflammatory cytokines [60]. After the hyperthermic period, hypothermia occurred during 24-72 h after cisplatin treatment. Hypothermia is reported in rats receiving chronic cisplatin treatment [61], but the underlying mechanism has not been identified. The hypothermic mechanism might be linked to the prolonged anorexia caused by cisplatin, as animals might lower body temperature to conserve energy during anorexia [62], but it could also be related to a marked reduction of activity which is noted to occur following cisplatin in the rat [63]. Central and peripheral administration of GLP-1 induces hypothermia lasting 2 h in rats, but the effect to reduce food intake is only seen following its central administration [64]. A continuous i.c.v. infusion of exendin-4 also caused hypothermia in mice [65], and blockade of central GLP-1 receptors enhanced LPS-induced fever in rats [66]. In the present studies, the continuous infusion of exendin (9-39) alleviated the hypothermia caused by cisplatin, suggesting a mechanism involving activation of central GLP-1 receptors. It is not known if the antagonism of the hypothermic action of cisplatin that we observed relate to the elevated BP during the delayed phase, where the mechanism could reflect a reduction of heat loss secondary to any possible vasoconstriction in the skin. Hypothermia has been proposed as a biomarker of motion sickness in rodents [67], but our failure to observe a consistent pattern during acute and delayed emesis indicates that caution must be exercised in any interpretation relating to ‘nausea’ alone. It is possible that inflammatory mediators that are presumed to be released during treatment with cisplatin may elevate temperature, which potentially masks any fall in temperature relating to ‘nausea’. In any event, it is important to note that the antagonism of hypothermia by exendin (9-39) did not impact on the profile of GMA and HRV that we recorded during the delayed phase of emesis.

### Food intake

Our previous acute experimentation in ferrets demonstrated that exendin-4 induces emesis, behaviours indicative of nausea (BIN), reduced food intake, hypertension, tachycardia, decreased HRV and a reduction of gastric slow wave power [24, 25, 68]. BIN and reduced food intake could occur independently of emesis or activation of the AP and NTS, whereas midbrain and forebrain activation were essential for BIN but not emesis, as assessed by c-Fos [25]. Conversely, the mechanism of cisplatin to reduce food intake in ferrets during the acute and delayed phases of emesis is markedly reduced by AP ablation [28]. Previous studies in *Suncus murinus* and ferrets have shown that exendin-4-induced emesis and BIN, but not the inhibition of food and water intake, can be reversed by pre-treatment with exendin (9-39) [5, 25]. These findings are consistent with the lack of effect of exendin (9-39) on cisplatin-induced reductions in food and water intake in the present study, and are comparable to studies in *Suncus murinus* [5].

In addition, a recent rat study showed cisplatin (6 mg/kg, i.p.) reduced of food intake (at 24 and 48 h) but exendin (9-39) (20 μg, i.c.v.) had no effect on the reduced food intake or weight loss at 24 h and only a marginal improvement at 48 h [69]. Overall, the data from three species is consistent in demonstrating that reduced food intake induced by cisplatin does not involve central GLP-1 pathways amenable to blockade by exendin (9-39).

The present study has for the first time simultaneously quantified gastric (GMA), cardiovascular (BP, HRV) and behavioural (food and water intake) effects of cisplatin, in addition to its emetic effects over the entire 72 h period encompassing acute and delayed emetic phases. The results provide novel insights into events that are likely to occur in patients undergoing anti-cancer chemotherapy. Exendin (9-39) reduced the emetic response by ~59 % during the acute phase providing evidence that GLP-1 receptors, previously demonstrated to be present in key brain regions implicated in emesis in the ferret [5], are present in the central pathway(s) resulting from the mechanisms (primarily abdominal vagal-enteroendocrine cell) activated during the acute phase of cisplatin-induced emesis [32, 70]. However, caution needs to be exerted in pursuing GLP-1 receptor antagonists as anti-emetics until the mechanism underlying the unexpected hypertensive effect of i.c.v. exendin (9-39) in the presence of cisplatin is identified. Cisplatin-induced anorexia does not appear to involve central GLP-1 receptors. Application of MFDFA to GMA data identified subtle differences in gastric effects of cisplatin between the acute and delayed phases of emesis which may have implications for treating the gastrointestinal toxicities of chemotherapy. It is unknown if the differences revealed by MFDFA contribute to the mechanism of delayed emesis or “nausea”. It may be possible that agents with a capacity to restore power and structure to slow waves may represent a novel class of drug with benefit against the side effects of chemotherapeutic challenges.

## MATERIALS AND METHODS

### Animals

Twelve castrated male fitch ferrets (1.53 ± 0.07 kg) were obtained from Southland Ferrets (Invercargill, New Zealand) and housed individually in observation cages (0.5 m x 0.5 m x 0.5 m) in a temperature-controlled room at 24 ± 1°C under artificial lighting, with lights on between 06.00 to 18.00 h. The relative humidity was maintained at 50 ± 5 %. Water and food (TriPro super premium chicken meal formula dog food, American Nutrition, USA) were given *ad libitum*, unless otherwise stated. All experiments were conducted under license from the Government of the Hong Kong SAR and the Animal Experimentation Ethics Committee, The Chinese University of Hong Kong.

### Transmitter implantation and stereotaxic surgery for infusion pump implantation

Animals were fasted overnight but allowed free access to water. They were then injected with buprenorphine (0.05 mg/kg, s.c. Temgesic®), and anaesthesia was induced by ketamine (20 mg/kg i.m.; Alfasan, Holland). They were intubated using a 2/0 tube, and anaesthesia was maintained with 1.5% isoflurane (Halocarbon Products Corporation, USA) in oxygen using an anaesthetic machine (Narkomed 2C, Drager, USA). Rectal temperature was monitored and maintained at 37°C using a heating pad (UCI#390 Analogue moist heating pad, Rebirth Medical & Design, Inc., Taiwan) and the level of anaesthesia was assessed and monitored throughout the surgery by the pedal withdrawal reflex. Following a midline abdominal incision, the catheter of a C50-PXT transmitter (Data Sciences, Inc., USA) was inserted into the abdominal aorta up to a length of approximately 2 cm. A 2 × 2 mm piece of sterile gauze was placed over the catheter's entry point, and fixed with a drop of tissue glue. The body of the transmitter was then sutured to the left side of the ferret's abdominal wall muscle with the biopotential wires and catheter facing caudally. The gastric antrum was exposed and the biopotential wires were inserted into the muscle and secured in place by serosal sutures. The abdominal cavity was sutured in closed layers. The head of the animals was then positioned and fixed into a stereotaxic frame equipped with custom-made ear-bars and mouthpieces (David Kopf Instruments, Tujunga, USA). The temporalis muscles were exposed via a skin incision and displaced exposing the skull. A hole was drilled in the skull: coordinates for the lateral ventricle: 17.3 mm anterior to lambda and 0 mm lateral to the midline. A 30-gauge cannula was then inserted into the hole 8 mm below the surface of the dura. Two screws were fixed on the skull and dental cement was applied on the two screws as well as on the guide cannula to fix the guide cannula. After fixation, a small incision was made at the back of neck, and the end outer tube of the iPRECIO™ pump (Primetech Corporation, Tokyo, Japan) was tunnelled underneath skin of the neck to the guide cannula. Then the end of the outer tube was connected to a custom-made bended 30 G needle, and the other end of the 30 G needle was inserted into the guide cannula with insertion depth identical to the length of guide cannula. The inserted needle was fixed by applying dental cement to the guide cannula and 30 G needle. After needle fixation, two anchors of the pump were sutured to the outside skin of the back of the neck by using 3/0 polypropylene suture (Prolene® Ethicon). Muscle and skin layers were closed respectively with continuous and discontinuous sutures of silk thread (2/0) (Mersilk®, Ethicon). The wound was sprayed with silicone spray dressing (Opsite®, Smith and Nephew, UK). Marbofloxacin (Marbocyl®, 2 mg/kg, s.c.) was administered once per day for 3 days and buprenorphine (0.05 mg/kg, s.c.) was given 8-12 h after the first dose. After surgery, animals were housed individually in observation cages with free access to food and water. They were allowed to recover for at least 7 days prior to further experimentation. Recovery was uneventful with no indications of infection; food and water intake were within the normal range.

### Drug treatment protocol

During the recovery period, animals were infused i.c.v. with saline at a flow rate of 3 μl/h. On the day of experiment, baseline telemetric data were recorded for 24 h. On the day of drug treatment, at 09.30 h, the saline from the pump reservoir was withdrawn, and then the pump was refilled with exendin (9-39) (100 nmol, 250 μl; the dose was based on our previous studies in ferrets showing anti-emetic and hypoglycaemic activity [25, 68]) or saline (250 μl). The programmed pump allowed the drug to start continuous infusion at 11.00 h with a flow rate of 10 μl/h. One hour after the start of drug infusion, cisplatin was injected at a dosage of 5 mg/kg, i.p. at 12.00 h; this dose has previously been established to reliably induce both acute and delayed emesis in the ferret [20]. After cisplatin injection, behaviour was recorded for 72 h via a video system (Panasonic WV-PC-240, China). Exendin (9-39) or saline was refilled twice at 24 and 48 h post cisplatin injection. Body weight and food and water intake were measured at 24 h intervals.

### Data analysis and statistics

Emesis was characterized as rhythmic abdominal contractions that were either associated with forceful oral expulsion of solid or liquid material from the gastrointestinal tract (i.e. vomiting), or not associated with passage of material (i.e. retching). Episodes of emesis (retching and/or vomiting) were considered separate when the animal changed its location in the observation cage, or when the interval between retches and/or vomits exceeded 5s [20].

Telemetric data were analysed using Spike2 (Version 7, Cambridge Electronic Design). The method for analysing GMA is described in previous studies; DP was defined as the highest power in the 0 to 15 cpm range, and DF was defined as the frequency bin with the highest power in the 0 to 15 cpm range [28]. Systolic BP was calculated from the peak of the blood pressure recording trace and diastolic BP was calculated from the trough. Mean arterial BP was defined as systolic BP/3+2^*^diastolic BP/3 [71]. For HR, the peak-to-peak interval was first calculated, and HR = 60/P-P interval (bpm). The time-domain HRV was calculated by taking the standard deviation of P-P intervals in 5 min segments [72]. The frequency-domain analysis was performed using the FFT. The total power of all R-R intervals in 5-min segments were determined, along with its LF (0.04-0.15 Hz) and HF (0.15-0.7 Hz) components and LF/HF ratio [73]. CBT was calculated by taking the average of the data per 5 min interval. All data were eventually averaged in 1 h periods for statistical analysis.

MFDFA of GMA data for each 24h epoch were also performed using Spike 2 following the methodology of [74] and [29]. In brief, the magnitude of the multifractality in the time series was estimated by the width of the spectrum Δα = αmax − αmin [31, 75]. 3D plots of GMA running spectrum analysis were created using OriginPro 9.1.0 (OriginPro 9.1.0, OriginLab Corporation, Northampton, USA). For additional details of MFDFA see Supplementary Materials.

All statistical analyses were performed using GraphPad Prism version 5 (GraphPad Prism version 5.0, Inc. California, USA). Differences in behaviour, food and water consumption, body weight, mean arterial BP, HR, HRV, CBT, GMA and Δα between baseline and post-cisplatin injection in saline groups were assessed using one-way ANOVA followed by a Bonferroni's multiple comparison tests, or by paired Student's t-tests, as appropriate. The difference between the parameters measured in saline and exendin (9-39) treated animals were assessed using repeated two-way ANOVA and Bonferroni tests. All data are expressed as mean ± SEM. Differences were considered statistically significant when *P*<0.05.

### Drug formulation

Exendin (9-39) amide (American Peptide Company, Sunnyvale, CA) was dissolved in saline (0.9% w/v). Cisplatin was purchased as a sterile saline solution at 1 mg/ml (David Bull Laboratories, Victoria, Australia) and injected in a volume of 5 ml/kg.

## SUPPLEMENTARY MATERIALS FIGURES AND TABLES


