# Supplementary material for: Gastric myoelectric activity during cisplatin-induced acute and delayed emesis reveals a temporal impairment of slow waves in ferrets: effects not reversed by the GLP-1 receptor antagonist, exendin (9-39)
Source: Oncotarget. 2017 Oct 16;8(58):98691–707. doi: 10.18632/oncotarget.21859 (PMC5716760; doi:10.18632/oncotarget.21859)
Supplement: Supplementary file 1 [file oncotarget-08-98691-s001.pdf]

Gastric myoelectric activity during cisplatin-induced acute and delayed emesis reveals a temporal impairment of slow waves in ferrets: effects not reversed by the GLP-1 receptor antagonist, exendin (9-39)

SUPPLEMENTARY MATERIALS

Steps of MFDFA analysis are described below:

Step 1: Computing the average

Let us suppose $x(i)$ for $i = 1 \ldots N$, be a non-stationary time series of length N. the mean of the above series is given by

$$x_{ave} = \frac{1}{N}\sum_{i=1}^{N} x(i) \qquad (1)$$

Step 2: Computing the integrated time series

$$Y_{(i)} \equiv \sum_{k=1}^{i} [x(k) - x_{ave}] \text{ for } i = 1 \ldots N \qquad (2)$$

Step 3: Dividing the integrated time series to N_s non-overlapping bins (where $N_s = int(\frac{N}{s})$ and s is the length of the bin), and computing the fluctuation function

$$F^2(s,v) = \frac{1}{s}\sum_{i=1}^{s} \{Y[(v-1)s+i] - y_v(i)\}^2$$

for each bin v, $v = 1, \ldots N_s$ and

$$F^2(s,v) = \frac{1}{s}\sum_{i=1}^{s} \{Y[N-(v-N_s)s+i] - y_v(i)\}^2$$

for $v = N_s + 1, \ldots, 2N_s$ where $y_v(i)$ is the least square fitted value in the *v*.

Step 4: Calculating the q-th order fluctuation and the scaling of the fluctuation of function:

$$F_q(s) = \{\frac{1}{2N_s}\sum_{v=1}^{2N_s} [F^2(s,v)]^{\frac{q}{2}}\}^{\frac{1}{q}} \qquad (3)$$

$$F_q(n) \propto n^{qh(q)} \qquad (4)$$

Where *h(q)* represents a generalized Hurst exponent. For multifractal time series the value of *h(q)* depends nonlinearly on q. From this point, the multifractal scaling exponent $\tau(q)$ can be calculated from *h(q)* by the relation:

$$\tau(q) = qh(q) - 1 \qquad (5)$$

The multifractal spectrum $f(\alpha)$ can be obtained from a Legendre transform of the $\tau(q)$ exponent:

$$\alpha = \tau'(q) \qquad (6)$$

$$f(\alpha) = q\alpha - \tau(q) \qquad (7)$$
